# Supplementary material for: Gesture recognition with Brownian reservoir computing using geometrically confined skyrmion dynamics
Source: Nat Commun. 2024 Sep 16;15:8103. doi: 10.1038/s41467-024-52345-y (PMC11405713; doi:10.1038/s41467-024-52345-y)
Supplement: Supplementary file 1 — Supplementary Information [file 41467_2024_52345_MOESM1_ESM.pdf]

Supplementary Information

S1. Reservoir Energy Efficiency

To estimate the energy consumption of a single device we examine the energy use of a scaled-down realistic version with a side length of $400\ \mathrm{nm}$ and a skyrmion size of $40\ \mathrm{nm}$ based on existing skyrmions stable at room temperature and that was previously used to analyze the power consumption[1,2]. At these dimensions, the ratio between the skyrmion and the device closely resembles that of the experimentally used $36\ \mu\mathrm{m}$ sized device. The device's resistance is calculated to be approximately $R = 1\ \mathrm{k}\Omega$ between the two connected contacts[2].

With a current density required to move the skyrmion of $J = 2.9 \times 10^{7}\ \mathrm{Am}^{-2}$ at half the triangle's width, we find a flow of up to $I = 64\ \mathrm{nA}$ through the device, including the $5\ \mathrm{nm}$ tantalum capping layer. Notably, as the capping layer serves as an anti-oxidation layer and does not impact the skyrmion dynamics, the required current could be reduced by nearly half with either connecting the device from the bottom or altering the capping layer material. Given these parameters, the worst-case power consumption of one module would be $P = RI^2 = 4\ \mathrm{pW}$, assuming continuous application of maximum voltage, while typically only a fraction of this is used in a typical signal.

Changing the skyrmion size also affects its dynamics and therefore reducing the time needed per input. On the one hand, the diffusion constant increases with smaller skyrmions[3] and on the other hand, the required path of the displacement becomes shorter.

To obtain the energy for the operation we first consider conservatively the low diffusion constant in the case of significant pinning as shown in the main text. This assumption yields a time per gesture of $t = 1.1\ \mathrm{ms}$. Consequently, we calculate an energy consumption of $E = Pt = 4\ \mathrm{fJ}$ per gesture. Which is comparable to conventionally used CMOS ring oscillators[4].

The free diffusion expected theoretically[5] and that we have shown experimentally can be generated by AC excitations[6] yields for these skyrmions a diffusion constant of $70 \times 10^{-9}\ \mathrm{m^2 s^{-1}}$ leading to a reduced time per operation of $780\ \mathrm{ns}$, resulting in ultra-low energy consumption of $3\ \mathrm{aJ}$ per gesture, significantly lower than the scaled down version of a previously proposed spin-torque nano-oscillators at $100\ \mathrm{aJ}$[7].

While we show that our device compares favorably to other reservoir computing devices, for the overall power consumption one needs to also consider the CMOS periphery. We emphasize that our device operates in quasi-dc regime and therefore has reduced demands for CMOS periphery as compared to oscillator based AC magnetic devices[7]. Hence, we expect that the CMOS overhead will reduce compared to the previously analyzed peripheral circuitry. For details of this power consumption, we refer to the pertinent literature[7,8].

In our architecture, the final layer incorporates a linear support vector machine (SVM). Recent progress has enabled the integration of SVMs into Field-Programmable Gate Arrays (FPGAs), facilitating an even more power-efficient implementation[9]. We note that a comparison with purely software-based solutions is not easily possible due to the complexity of state-of-the-art used CPUs or Cloud solutions and such an analysis comprises research that goes clearly beyond the scope of this work.

S2. Readout Layer and Total Energy Efficiency

When comparing the reservoir readout layer with the pure software-based approach using a linear SVM, both methods require the same computational complexity and, consequently, the same amount of energy. However, when comparing the readout with multiple MTJs to the non-linear radial basis SVM (RBF SVM on RD data), the reservoir demonstrates a significant advantage in terms of energy consumption for both inference and training.

To validate the high energy consumption of the RBF SVM, we have measured the inference time of the reservoir's readout layer (LIN SVM on a 5 MTJ Reservoir) and the pure software-based approach (RBF SVM on RD data) (see Figure 4). The RBF SVM's inference time increases polynomially with the dataset size, whereas the reservoir's linear readout maintains a constant inference time. Notably, the reservoir's inference time decreases with larger dataset sizes, as the "warm-up" time becomes negligible in comparison.

We also plotted the best and worst-case scenarios for the reservoir readout, which vary with the MTJs' temporal resolution. This parameter is optimized for better detection accuracy and affects the dimensions of the readout layer. Our observations show that the reservoir readout reduces the required time by a factor of three compared to the RBF SVM with the smaller dataset used in the experiment, with an even greater time reduction for larger datasets.

Since the inference time is directly proportional to energy consumption, we demonstrate a significant improvement in energy efficiency when using the reservoir readout compared to the RBF SVM. Additionally, the total energy consumption of the reservoir is lower because the energy required to operate the reservoir is insignificant compared to the readout layer. To validate this, we can assume that the software-based approach consumes about $100\ \mathrm{W}$ during operation. With the given inference time, this results in $350\ \mathrm{uJ}$ for the RBF SVM and $90\ \mathrm{uJ}$ for the reservoir readout layer in the worst case. Compared to the energy required for the reservoir, which is $48\ \mathrm{pJ}$, this is not significant.

Supplementary References

1. Everschor-Sitte, K., Masell, J., Reeve, R. M. & Kläui, M. Perspective: Magnetic skyrmions—Overview of recent progress in an active research field. *J. Appl. Phys.* **124**, 240901 (2018).
2. Raab, K. *et al.* Brownian reservoir computing realized using geometrically confined skyrmion dynamics. *Nat. Commun.* **13**, 6982 (2022).
3. Zázvorka, J. *et al.* Thermal skyrmion diffusion used in a reshuffler device. *Nat. Nanotechnol.* **14**, 658–661 (2019).
4. Saheb, Z., El-Masry, E. & Bousquet, J.-F. Ultra-low voltage and low power ring oscillator for wireless sensor network using CMOS varactor. in *2016 IEEE Canadian Conference on Electrical and Computer Engineering (CCECE)* 1–5 (2016). doi:10.1109/CCECE.2016.7726620.

5. Miltat, J., Rohart, S. & Thiaville, A. Brownian motion of magnetic domain walls and skyrmions, and their diffusion constants. *Phys. Rev. B* **97**, 214426 (2018).
6. Gruber, R. *et al.* 300-Times-Increased Diffusive Skyrmion Dynamics and Effective Pinning Reduction by Periodic Field Excitation. *Adv. Mater.* **35**, 2208922 (2023).
7. Romera, M. *et al.* Vowel recognition with four coupled spin-torque nano-oscillators. *Nature* **563**, 230–234 (2018).
8. Mizrahi, A. *et al.* Neural-like computing with populations of superparamagnetic basis functions. *Nat. Commun.* **9**, 1533 (2018).
9. Afifi, S., GholamHosseini, H. & Sinha, R. FPGA Implementations of SVM Classifiers: A Review. *SN Comput. Sci.* **1**, 133 (2020).
